# Service quality assessment of a referral hospital in Southern Iran with SERVQUAL technique: patients’ perspective

**DOI:** 10.1186/1472-6963-14-322

**Published:** 2014-07-27

**Authors:** Teamur Aghamolaei, Tasnim Eghbal Eftekhaari, Shideh Rafati, Kobra Kahnouji, Shamsieh Ahangari, Mohammad Esmaeil Shahrzad, Ataollah Kahnouji, Seyedeh Hamideh Hoseini

**Affiliations:** 1 Department of Public Health, Social Determinants for Health Promotion Research center, Hormozgan University of Medical Sciences, Bandar Abbas, Iran; 2 Shahid mohammadi Hospital Clinical Research Development Unit, Hormozgan University of Medical Sciences, Bandar Abbas, Iran; 3 Social Determinants for Health Promotion Research Center, Hormozgan University of Medical Sciences, Bandar Abbas, Iran; 4 Department of Management, University of Sistan and Baluchistan, Zahedan, Iran; 5 Research Committee, Hormozgan Universtiy of Medical Sciences, Bandar Abbas, Iran; 6 Islamic Azad University of Rafsanjan, Rafsanjan, Iran; 7 Health information management Research Center, Hormozgan University of Medical Sciences, Bandar Abbas, Iran

**Keywords:** Hospital, Quality, SERVQUAL, Patients, Iran

## Abstract

**Background:**

Providing services to patients according to their expectations and needs is necessary for the success of an organization in order to remain in the competitive market. Recognizing these needs and expectations is an important step in offering high quality services. This study was designed to determine the service quality gap of the main hospital of Hormozgan province.

**Methods:**

This cross sectional study was conducted in 2013 in Bandar Abbas ShahidMohammadi Hospital in the south of Iran. All 96 participants of this study were provided by SERVQUAL questionnaire. Data was analyzed by Wilcoxon and Kruskal-Wallis tests.

**Results:**

Service quality gaps were seen in all five service quality dimensions and the overall quality of service. The mean of quality perception score and quality expectation score was 3.44 ± 0.693 and 4.736 ± 0.34, respectively. The highest perception was in assurance dimension and the highest expectation was in Responsiveness and assurance dimensions. Also, the lowest perception was in responsiveness dimension and the lowest expectation was about empathy. In this study, 56.1% of participants defined the quality of services as average.

**Conclusion:**

According to the results, this hospital was not able to meet patients’ expectations completely. Therefore, action must be taken to decrease the gap between the perception and expectation of the patients.

## Background

Nowadays, quality is becoming a burden to organizations in order to satisfy their customers. Quality of services is an important factor for the growth, success and persistence of an organization and is becoming an important factor for forecasting the organizations’ perspective [1].

In the health sector the importance of health services and their relation with human life, quality assurance and quality promotion have increasingly caught the attention of tax payers having increasing expectations from hospitals and other health providing organizations. Because of the importance of health care services, improving their quality is becoming more and more substantial and the demand for quality control and quality management is increasing [2].

Quality is a familiar term that is used in many settings. Compatibility between the service and what the customer needs and expects is the most common definition [3]. Quality is achieved when the service meets customer’s needs and expectations [4]. Any attention to the service, without counting on customers’ opinion will not improve quality, necessarily. Accordingly, inquiring customer’s opinion for adapting the services is important [4].

The quality of health services has two dimensions; technical quality and functional quality [5]. The technical quality of health care services is on the bases of the authenticity of identification and management procedures. On the other hand, functional quality is related to non-clinical aspects [6].

Since most of the patients have no knowledge towards the assessment of technical services, the functional quality is used to evaluate the quality of healthcare services [5]. “In order to assure that medical procedures are effective not only from the expert’s viewpoint (technical quality) but also having the ability to satisfy the functional quality patients expectations must be considered in health system delivery; hence it is essential to evaluate services explicitly and implicitly based on consumer’s viewpoints” [6]. Providing the services according with patient’s needs and expectation is essential for survival and success of the organization in the competitive environment of the health care market [7].

The expectations of consumers are related to their previous expectations, attitude and perception towards the competitive markets. Also, after providing services the organization managers must measure the amount of expectations that have been met [8].

Usually there is an imbalance between the patients’ real needs and discernments and awareness of managers from perceptions of the patients which decreases the quality of services [9].

(One of the most important reasons is that direct relation with the customer is not available. Therefore the managers will not be able to determine the priorities and cannot answer the expectations which lead to quality gaps [10].

However, recognizing the expectations and perceptions of costumers regarding the quality of services will facilitate prioritizing and strategic resource allocation and will decrease quality gaps [11].

There are different methods for determining the expectations of patients. SERVQUAL is one of the best and most used models in this regard [12]. This questionnaire was reviewed many times and is now summarized in 5 dimensions of perceptions: responsiveness, assurance, reliability, empathy and tangibility [13].

This tool is used in order to measure the quality of service, which has 5 dimensions and 22 components for measuring the expectations and perceptions of the patients on the dimensions of the service qualities [14].

SERVQUAL assesses the exact insight of the patients from the services they receive and compares it with their ideal expectation. Parasurman believes that the quality of services is related to their expectations before and during purchasing and its perceived quality after purchasing. He also defines service gap as the difference between customer expectation and their perceptions. This model is also recognized as the gap analyzer model and is the strongest tool in assessing the quality of services [15].

This study follows the aim of determining the different dimensions of the quality of the services being provided in ShahidMohammadi hospital in south of Iran and evaluating service quality from the patients’ perspective.

## Methods

The study population consisted of all the patients who were hospitalized in ShahidMohammadi Hospital of Bandar Abbas in south of Iran during the spring of 2013. Only children under age of 15 yrs were admitted because of surgical, orthopedic or neurosurgical problems or major burns. The patients were assured their information would be kept confidential and an informed consent was obtained. For the children younger than 15 years the consent was filled out by their guardians or parents. This hospital has 21 wards and 450 beds.

### Sampling

The sample size was calculated according to the results of other studies. With a maximum error of 0.05, standard deviation of 0.22 and confidence level of 95%, the sample size was 97. The samples were selected using multistage cluster method. At first, all the hospital wards were listed and among them, eight wards were selected. Then, according to the size of each cluster, the samples were selected from patients who were hospitalized for more than 24 hours. A total of 100 questionnaires were distributed among the patients on the day of discharge and filled by an interviewer. Out of 100questionnaires, 11 were excluded from the study due to incompleteness.

### Survey instrument

The required data were collected using Parasuraman questionnaire [12]. This questionnaire consists of a part with demographic questions and another part with 22 multiple choice questions; four questions related to tangibility, four questions related to responsiveness, five questions related to reliability, four questions related to assurance and five questions related to empathy. In this part, the expectations and perceptions of patients were measured. In the first section, the perception of patients of the quality of services is measured and in the second section, their expectations of quality of services is assessed using Likert scale ranging from strongly disagree (1) to strongly agree (5) to assess the level of patients’ expectation and perception of service quality. To classify the perception and expectation of the patients from the hospitals’ service quality in each of the 5 dimensions, perceptions was divided to good, intermediate and weak; expectation was divided to very important, relatively important and least important (Table 1) according to the total score of questions in each dimensions. So that maximum score in that dimension was subtracted from minimum score in that dimension, and added by one and divided by three. The resulted number showed the array of the groups.

**Table 1:** The state of perception and expectation of participants of the study

	**Perception**						**Expectation**			
	**Strong**		**Moderate**		**Weak**		**Very important**		**Relatively important**	
	**N**	**%**	**N**	**%**	**N**	**%**	**N**	**%**	**N**	**%**
Tangibility	29	32.6	51	57.3	9	10.1	86	96.6	3	3.4
Reliability	32	36	52	58.4	5	5.6	86	96.6	3	3.4
Responsiveness	34	38.2	48	53.9	7	7.9	87	97.8	2	2.2
Assurance	39	43.8	43	48.3	7	7.9	86	96.6	3	3.4
Empathy	26	29.2	56	62.9	7	7.9	84	94.4	5	5.6
Total quality	32	36	50	56.1	7	7.9	86	96.6	3	3.4

The quality gap was defined as the difference of the perception and expectation scores.

The validity of the Persian translation of the questionnaire was confirmed by experts and had been previously used by many Iranian researchers. The Reliability of the questionnaire was also confirmed (Cronbach’s α = 0.94).

### Analysis

Collected data was entered in SPSS ver19 software. Wil-coxon test was used to calculate the quality gap of services and Kruskal-Wallis was used to define the significance of difference between the mean score of expectation and perception in different age, gender, educational status, and employment groups.

### Ethics

This study was approved by the ethics committee of the Deputy of Research and Technology, Hormozgan University of Medical Sciences (code: HEC-92-12-14).

## Results

The mean age of the participants was 32.9 ± 10.05 and most of the participants were male (64%). Most of them were unemployed (58.4%) and the education level of 55.1% was below high school.

As shown in the Table 1 based on the perceptions of the patients, 56.1% of the participants recognized the total quality as average and Based on their expectations 96.6% of the patients believed that quality was the most significant aspect of services. Also, none of the patients recognized any of the services as not important (96.6%) (Table 1).

Based on our findings, the mean expectation score was high. It was 4.87 for the cleanliness status and 4.62 for the neat and well-dressed personnel status of hospital employees. Among the five dimensions of quality, the highest expectation score was related to responsiveness and assurance (4.76) and the lowest score was related to empathy (4.69 ± 0.47). The mean score of perception varied from 3.92 (neat employees and well-dressed personnel) to 3.02 (cleanliness of hospital environment). The highest perception score was associated with assurance among all of the dimensions (3.56 ± 0.86). And in the next order were reliability, tangibility, empathy, and responsiveness.

The quality gap of the services was calculated and Wilcoxon test showed that the difference between the expectation and perception of the patients was statistically significant in all of dimensions. And hence, there is a gap between the patients ’perception and their expectation of the service quality of ShahidMohammadi hospital (P < 0.001) [Table 2].

**Table 2:** Mean score of perception, expectation and quality gap of services provided by Shahid Mohammadi Hospital

	**Perception**	**Expectation**	**Quality gap**	**Z**	**P value**
Tangibility	3.42 ± 0.83	4.73 ± 0.40	-1.30 ± 0.96	-7.73	<0.001
Reliability	3.49 ± 0.72	4.72 ± 0.43	-1.22 ± 0.87	-7.72	<0.001
Responsiveness	3.34 ± 0.81	4.76 ± 0.38	-1.42 ± 0.91	-7.81	<0.001
Assurance	3.56 ± 0.86	4.76 ± 0.47	-1.20 ± 0.96	-7.78	<0.001
Empathy	3.39 ± 0.8	4.69 ± 0.47	-1.31 ± 0.96	-7.78	<0.001
Total quality	3.44 ± 0.69	4.73 ± 0.34	-1.29 ± 0.81	-8.06	<0.001

Our findings showed that the highest quality gap was related to the responsiveness dimension (1.426) while the lowest gap of quality was related to the assurance (1.202) [Table 2].

Kruskal-Wallis test showed that age, gender, education level and employment status had no relationship with the quality of hospital services.

## Discussion

This study was designed to determine the quality gap of services according to the perception and expectations of patients in an educational hospital in south of Iran, furthermore to aid health policy makers in suitable programming for appropriate hospital medical services.

According to the patients’ perceptions the quality of services in ShahidMohammadi hospital was in average. The lowest perception was related to correct performance of the services at the first time, willingness of personnel to help patients, attending of personnel whenever called, telling when services will be performed; that was constituent responsiveness dimension. Which was parallel with results of Lau’s study in Malaysia [16] and Lim’s study in Singapore [17].

Also the highest expectation score was related to responsiveness dimension. Since the highest expectations and the lowest perceptions was in responsiveness dimension and that low perceived responsiveness threatens the hospital’s ability to achieve patients satisfaction, if the hospital is looking for improvement of hospital services and increasing the patients satisfaction, in the first instance training of staff on patient requirements is essential. And it is also recommended that hospital authorities pay more attention towards patients’ rights and be more responsible against them. Therefore hospital staffs especially those who are in direct contact with the patients and have the most significant effect on the hospital service quality and the patients’ satisfaction, should get enrolled in the service quality improvement programs.

According to patients’ perception, assurance was the most desirable dimension of service quality in this study. This result was consistent with the results of Lim and Tang and Karydis et al. [17,18]. In a study by Aghamolaei al, the most important dimension of the service quality was found to be assurance, followed by responsiveness and empathy [19]. Since assurance is related to the instilling confidence in patients, feeling safety and security in interaction with personnel, existence of knowledgeable personnel to answer patients’ needs, and polite and friendly dealing of personnel with patients, It can be concluded that according to patients’ belief in this medical training center, physicians and employees have sufficient knowledge to manage the patients, and with their courteous behavior, cause the patients to feel secure and safe.

Also the results showed that the lowest expectation was related to empathy and the highest expectation was related to responsiveness and assurance dimensions. Moreover the results showed that the expectation of patients is high. This part of results is parallel with a study conducted by Lim and Tang and other studies by Zarei et al. and Ranjabarezatabadi et al. [17,8,20].

As the results showed, in all of domains of services, patients’ expectations of the services provided were higher than their perceptions and the gaps between patients’ perceptions and their expectations were negative. The highest negative gap was in responsiveness dimension and the lowest negative gap was in assurance dimension. The negative gaps indicate that patients’ expectations of the services provided are higher than their perceptions.

Many researchers have tried to assess the gap between expectations and perception of costumers regarding the services. Caha in a private hospitals in Turkey [21], Yesilada and Direktor in general hospitals of Cyrus [22], Nekoei Moghadam and Amiresmaeili in educational hospitals of Kerman [6], Tabibi in outpatient clinics of Tehran [23], and Ranjbarezatabadi in ShahidSadughi hospital of Yazd [20] reported negative gaps in some dimensions of SERVQUAL. Ranjbarezatabadi reported that tangibility and responsiveness dimensions had the highest gaps, while assurance and empathy had the lowest gap.

Today quality is defined as “the demands of customers” and customers’ perceptions and expectations are the main elements of quality [24,25]. Hence, responding the customers’ expectations has an important role in improving the quality of services and increasing the satisfaction of costumers.

Our study had some limitations that restrict the generalization of the results. The findings are based on the results of one referral hospital in Hormozgan province, located in south of Iran, so other studies must be piloted in other parts of the Hormozgan province and private hospitals in Bandar Abbas to increase the generalizability of results of this study. Another limitation was the small number of patients which could not be generalized to Hormozgan province’s actual population for estimation of actual hospital service quality.

## Conclusion

The results of this study showed that the quality of services is in average and there were negative gaps between patient’s perceptions and expectations in all of dimensions of service quality provided. So, to achieve the ideal level, proper planning and effective efforts should be implemented.

## Competing interests

The authors declare that they have no competing of interest.

## Authors’ contributions

TA: Developed the concept. Designing the study. TEE: Technical and Scientific editing of the manuscript. SR: Analyzed the Data. KK: Developed the Main Concept, interpreted the data. Writing the manuscript SA: Reviewed the literature. MES: Translated the manuscript. Review of the literature. AK: preparing the draft. SHH: Data collection. All authors read and approved the final manuscript.

## Pre-publication history

The pre-publication history for this paper can be accessed here:

http://www.biomedcentral.com/1472-6963/14/322/prepub

## References

[B1] Sangeeta S Banwet DK Karunes S An integrated framework for quality management in education: a faculty perspective TQM J 2008 14 5 502 519

[B2] Tabibi SJ EbadiFardeAzar F Khalesi N Torani S Total Quality Management In Health Care System 2001 Tehran: JahanRayane Press 2 3 [Book in Persian]

[B3] Fitz Simmons JA Fitz Simmons M Service Management, Strategy, Operations and Information Technology 2001

[B4] Sadigh SM Service quality in hospitals: more favourable than you might think Manag Serv Qual 2003 14 3 197 206

[B5] Lam SSKSERVQUAL A tool for measuring patients” opinions of hospital service quality in Hong Kong Total Qual Manag 1997 14 4 145 152

[B6] Nekoei-Moghadam M Amiresmaili MR Hospital services quality assessment: Hospitals of Kerman University of Medical Sciences, as a tangible example of a developing country Int J Healthc Qual Assur 2011 14 57 66 10.1108/09526861111098247 21456498

[B7] Delene LM Bunda MA Kim C Methods of measuring health-care service quality J Bus Res 2000 14 233 246

[B8] Zarei A Arab M Rahimi Froushani A Rashidian A Ghazi Tabatabaei M Service quality of private hospitals: the Iranian Patients’ perspective BMC Health Serv Res 2012 14 31 22299830 10.1186/1472-6963-12-31 PMC3306759

[B9] Donnelly M Wisniewski M Dalrymple JF Curry AC Measuring service quality in local government: the SERVQUAL approach Int J Public Sec Manag 1995 14 7 15 20

[B10] Sewell N Continuous quality improvement in acute health care: creating a holistic and integrated approach Int J Health Care Qual Assur 1997 14 1 20 26 10.1108/09526869710159598 10166023

[B11] Parasuraman A Zeithaml VA Berry L A Conceptual model of service quality and its implications for future research J Mark 1985 14 41 50

[B12] Parasuraman A Zeithaml VA Berry LL SERVQUAL: a multiple-item scale for measuring consumer perceptions of service quality J Retail 1988 14 1 12 40

[B13] Berry LL Parasuraman A Zeithaml VA Ten Lessons for Improving Service Quality. Marketing Science Institute Report 1993 93 104

[B14] Zeithaml VA Bitner MJ Service Marketing 1996 New York: Mc Graw Hill

[B15] Brooks R Linggs I Botschen M International marketing and customer driven wave fronts Serv Ind J 1999 14 4 49 67

[B16] Lau P Khatibiakbar A Young Gun D Service quality: a study of the luxury hotels in Malaysia J Am Acad Bus 2005 14 2 10 46

[B17] Lim PC Tang N A study of patient’s expectations and satisfaction in Singapore hospitals Int J Health Care Qual Assur 2000 14 7 290 299 10.1108/09526860010378735 11484647

[B18] Karydis A Komboli M Pannis V Expectation and perception of Greek patients regarding the quality of dental health care Int J Qual Health Care 2001 14 5 409 416 11669569 10.1093/intqhc/13.5.409

[B19] Aghamolaei T Zare SH Poudat A Kebriyaei A Customers perception and expectation of primary health care services quality in health centers of Bandar Abbas Hormozgan Univ Med Sci J 2007 14 173 179 [Persian]

[B20] Ranjbarezatabadi M Bahrami M ZareAhmadabadi H Arab M Nasiri S Hataminasab H Analysis of SERVQUAL in Shahid Sadoghi hospital, Yazd, Iran Hormozgan Univ Med Sci J 2012 14 4 333 340

[B21] Caha H Service quality in private hospitals in Turkey J Econ Soc Res 2007 14 55 59

[B22] Yesilada F Direktör E Health care service quality: acomparison of public and private hospitals Afr J Bus Manage 2010 14 962 971

[B23] Tabibi SJ Gohari MR Shahri S Aghababa S Assessment of Health Care Services in outpatient clinics based on SERVQUAL model in Hospitals of Tehran Payavard Salamat 2012 14 4 49 56

[B24] West E Management matters: the link between hospital organisation and quality of patient care Qual Health Care 2001 14 1 40 48 11239143 10.1136/qhc.10.1.40 PMC1743422

[B25] Sharma B Gadenne D An investigation of the perceived importance and effectiveness of quality management approaches TQM Mag 2001 14 6 433 445

